# Past and future trends of *Cryptosporidium in vitro* research

**DOI:** 10.1016/j.exppara.2018.12.001

**Published:** 2019-01

**Authors:** Alexander J. Bones, Lyne Jossé, Charlotte More, Christopher N. Miller, Martin Michaelis, Anastasios D. Tsaousis

**Affiliations:** a Laboratory of Molecular and Evolutionary Parasitology, RAPID Group, School of Biosciences, University of Kent, Canterbury, Kent, UK; b School of Biosciences, University of Kent, Canterbury, Kent, UK

## Abstract

*Cryptosporidium* is a genus of single celled parasites capable of infecting a wide range of animals including humans. *Cryptosporidium* species are members of the phylum apicomplexa, which includes well-known genera such as *Plasmodium* and *Toxoplasma*. *Cryptosporidium* parasites cause a severe gastro-intestinal disease known as cryptosporidiosis. They are one of the most common causes of childhood diarrhoea worldwide, and infection can have prolonged detrimental effects on the development of children, but also can be life threatening to HIV/AIDS patients and transplant recipients. A variety of hosts can act as reservoirs, and *Cryptosporidium* can persist in the environment for prolonged times as oocysts. While there has been substantial interest in these parasites, there is very little progress in terms of treatment development and understanding the majority of the life cycle of this unusual organism. In this review, we will provide an overview on the existing knowledge of the biology of the parasite and the current progress in developing *in vitro* cultivation systems. We will then describe a synopsis of current and next generation approaches that could spearhead further research in combating the parasite.

## Highlights

• The biology and life cycle of *Cryptosporidium*.
• The *status quo* on *Cryptosporidium* culturing.
• New era on *Cryptosporidium* ‘omics.
• *Cryptosporidium* drug screening and potential treatments.

Organisms comprising the genus *Cryptosporidium* ssp. were first identified by Tyzzer in 1907, who discovered *Cryptosporidium muris* in the gastric glands of laboratory mice, but it was nearly 70 years before *Cryptosporidium* spp. were identified as pathogens in humans (Nime et al., 1976). In the 1980s, when AIDS was becoming more widespread, it also became apparent that *Cryptosporidium* was prevalent among the immunocompromised (Navin and Hardy, 1987). Since then, *Cryptosporidium* species have been identified as pathogens in humans, livestock, domestic pets and wild animals. The organisms are frequently found in the gastrointestinal tract (Noordeen et al., 2002); however, the precise location varies between species. Cryptosporidiosis usually presents as acute, non-bloody watery diarrhoea, but symptoms can include nausea, vomiting and fever, among others (DuPont et al., 1995; Chappell et al., 2006, 2011). While the disease is self-limiting in otherwise healthy adults (DuPont et al., 1995; Chappell et al., 2006, 2011), the infection can be chronic, and fatal, in the immunocompromised humans (Weisburger et al., 1979) and young children (Checkley et al., 2015) and animals (Pozio et al., 1997; Matsuura et al., 2017). In addition, children can experience nutrient malabsorption and growth retardation when infection becomes chronic, even if no symptoms are present (Tumwine et al., 2003; Bushen et al., 2007). There is only one FDA-approved drug treatment for cryptosporidiosis, nitazoxanide, which resolves diarrhoea after three or four days of treatment in most immunocompetent individuals (Rossignol et al., 2001; Amadi et al., 2002), but shows little efficacy in young children and immunocompromised individuals such as HIV/AIDS patients (Amadi et al., 2002). Despite the worldwide prevalence of *Cryptosporidium*, there remain a number of unanswered questions regarding its complex and multi-staged life cycle, in particular the molecular signals, which determine progression through the various stages and the precise mechanism of pathogenicity. A major factor preventing further understanding is still the lack of reliable, prolonged, *in vitro* models, which can accurately reproduce the *in vivo* life cycle (Hijjawi, 2010). This review will explore the cell biology of *Cryptosporidium* as well as the current progress in *in vitro* culture and ‘omics investigations, before exploring the future challenges of cryptosporidiosis.

## 1 *Cryptosporidium*: endogenous growth and life cycle

**Fig. 1:**
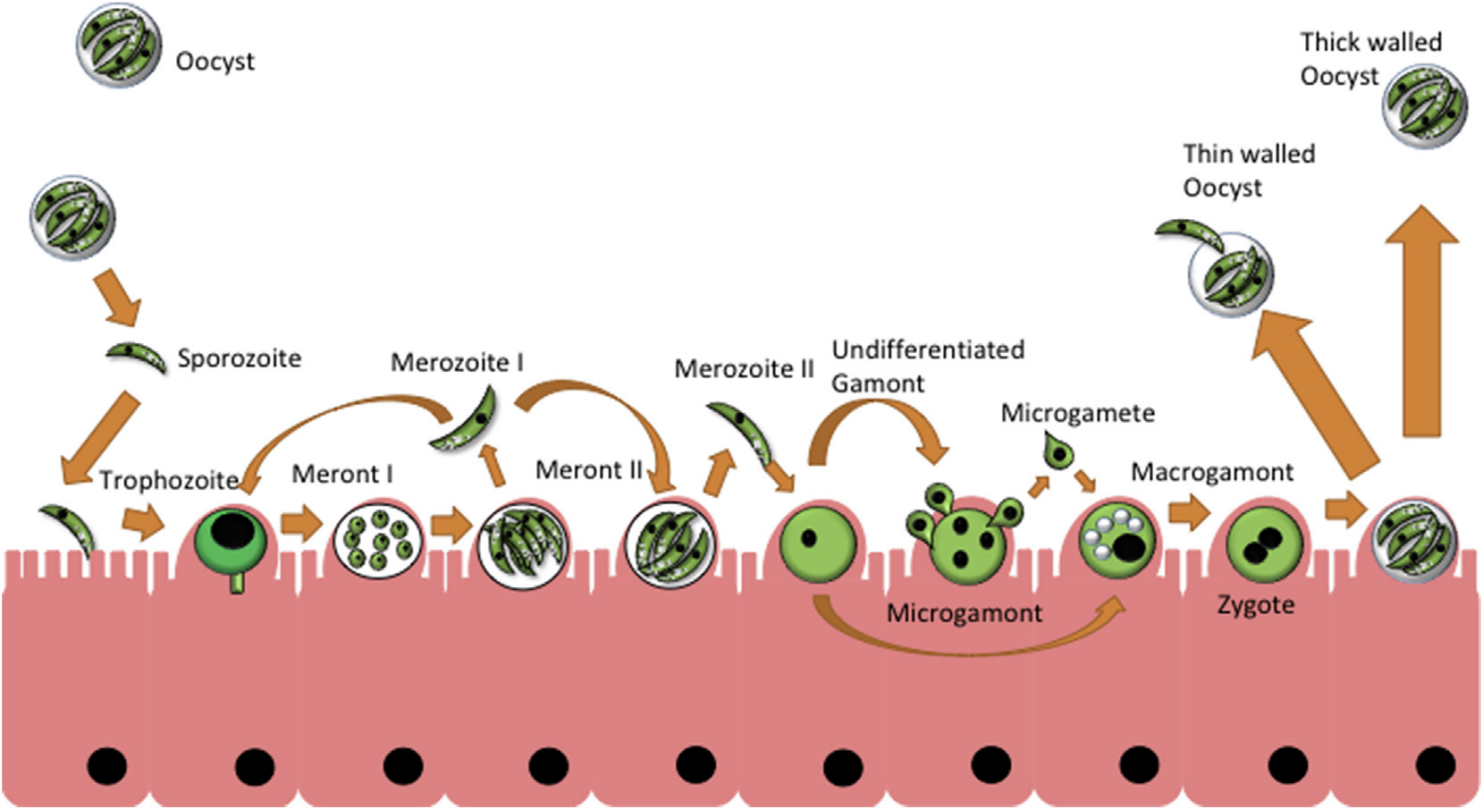
**Outline of the *Cryptosporidium parvum* life cycle** [Adapted and modified from (Thompson et al., 2005)]. Ingested oocysts release sporozoites, which invade the ileum, developing into trophozoites and type I meronts, containing type I merozoites. Released type I Merozoites can become trophozoites themselves or develop into type II meronts, which release type II merozoites, these develop into undifferentiated gamonts. Gamonts differentiate into macrogamonts or microgamonts, the latter produces microgametes, which fertilise macrogamonts. Sporulation occurs within the host, releasing thick walled oocysts into the environment and thin walled oocysts, which auto-infect the same host organism.

*Cryptosporidium* is widely considered to be an intracellular parasite, and occupies an intracellular, but extra-cytoplasmic vacuole within the host (Beyer et al., 2000; Petry, 2004; O'Hara and Chen, 2011). The life cycle consists of numerous stages, and is usually divided into an initial, asexual portion followed by the sexual stages, outlined in Fig. 1. *Cryptosporidium* species are monoxenous, completing their entire life cycle within a single host. Transmission occurs by ingestion of oocysts. *C. parvum, C. meleagridis* and *C. hominis* oocysts are small, circular bodies measuring 3–6 μm in diameter. Within the oocyst are four sporozoites measuring 2 μm × 0.8 μm. Thick walled *C. parvum* oocysts are extremely hardy and are resistant to many common disinfection methods (Barbee et al., 1999). *C. hominis* oocysts seem to share similar sensitivity to UV radiation, exhibiting 90% inactivation at 3 mJ/cm^2^ UV (Johnson et al., 2005). *C. parvum* oocysts stored between 0 and 20 °C are infectious for several days (Fayer and Nerad, 1996) and, in some cases up to 24 weeks (Fayer et al., 1998; Noordeen et al., 2002). Inactivation has been reported to be achieved by heating to 55 °C, and boiling for 5 min being adequate for inactivation (Fujino et al., 2002). The highly resistant nature of the oocysts has been attributed to a complex lattice structure (Harris and Petry, 1999), a surface glycocalyx layer (Liu et al., 2010), carbohydrates, fatty acids and aliphatic hydrocarbons, hydrophobic proteins and an inner glycoprotein layer (Jenkins et al., 2010). Transcriptomic analysis has revealed that oocysts are highly active in protein synthesis and translation, along with possessing an active proteasome, and ubiquitin machinery (Zhang et al., 2012). As *C. parvum* lacks all *de novo* nutrient synthesis genes and oocysts become less infectious with age (Slifko et al., 1997), it is likely that the parasite relies on protein recycling and its own reserves of amylopectin to survive shifts in environmental conditions (Fayer, 1994; Zhang et al., 2012). *Cryptosporidium* oocysts continued persistence in the environment, combined with high shedding; e.g. 6 × 10^11^ oocysts during the first year of life in *C. parvum* infected calves (Uga et al., 2000) show the potential health threat of these organisms.

Ingestion of an oocyst causes a process known as excystation, expansion of a “suture” (opening in the oocyst wall) and release of four motile sporozoites (Reduker et al., 1985a, 1985b). Sporozoites exhibit gliding motility (Forney et al., 1998; Wetzel et al., 2005). *In vitro* attachment and invasion occurs preferentially at 37 °C and at a pH of 7.4–7.6 (Chen et al., 1998; Huang et al., 2004), which are the conditions of the distal ileum (Evans et al., 1988), where infection is observed frequently. The sporozoite attaches to the plasma membrane of a host cell and is then enveloped (Chen et al., 1998), followed by transformation into a replicative trophozoite. Notable morphology changes include the parasite becoming rounded and enlargement of the nucleus (Bonnin et al., 1999; Beyer et al., 2000). Internalisation of the parasite recruits vacuoles to the infected location, causing fusion and formation of the parasitophorous vacuole (Huang et al., 2004). The transformation from invasive sporozoite to replicative trophozoite involves both host and parasite derived factors (O'Hara and Chen, 2011). Glycoproteins present in sera, unidentified factors secreted by host cells and Gal-GalNAc are all capable of inducing transformation into trophozoites (Edwinson et al., 2016).

**Fig. 2:**
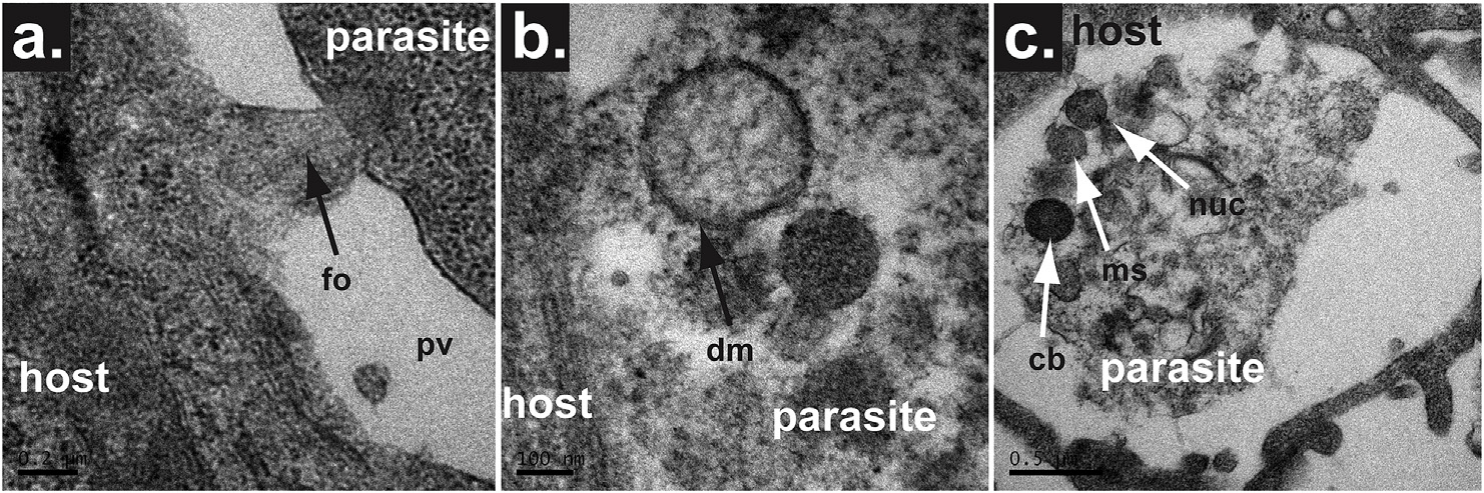
**Organelles in *C. parvum* infection.** Transmission Electron Microscopy (TEM) images showing the presence of a feeder organelle and mitochondrial-related organelles that are formed within the parasite infecting COLO-680N cells. **a.** TEM image showing the formation of the feeder organelle (fo), which anchors the main body of the parasite to the peripheral of the parasitophorous vacuole (pv). **b.** TEM image demonstrating a clearly identifiable double membrane structure of the mitosome within a merozoite form of *C. parvum*. **c.** TEM image showing numerous organelles within the sporozoite that has recently invaded a host cell. The electron dense structure that can be seen is the crystalloid body (cb). The nucleus (nuc) and the mitosome (ms) can also be seen in this image.

Trophozoite development is accompanied by the formation of the ‘feeder organelle’, a tube or tunnel which connects the parasite to the host cell cytoplasm (Bonnin et al., 1999; Beyer et al., 2000; Huang et al., 2004; Umemiya et al., 2005) (Fig. 2a). The feeder organelle has been hypothesised as a mechanism of myzocytosis engaged in by other gregarines, however no studies have shown transfer of host cytoplasm to the parasite. The ATP binding cassette protein CpABC-1 is localised to the host-parasite interface (Perkins et al., 1999; Zapata et al., 2002) and likely confers selective nutrient uptake and/or metabolite expulsion. Beyer and colleagues demonstrated that replicative stages remain intracellular, but extracytoplasmic in rat ileal cells (Beyer et al., 2000). However, the parasite can also be found within the cytoplasm of intraepithelial macrophages *in vivo*. The life cycle progresses through rounds of asexual merogony followed by fertilisation of gamonts, development of zygotes and oocyst production. Trophozoites divide by merogony to generate a type I meront containing six to eight type I merozoites, which when released continue this cycle by developing into trophozoites upon invasion.

At some stage, certain type I merozoites will develop into type II meronts, which contain four type II merozoites (Fig. 1). This starts the sexual stage of multiplication, as these merozoites develop into undifferentiated gametes upon cell invasion, and will then develop into macrogamonts or microgamonts, the latter of which produces microgametes. The gametes fertilise the macrogamont and form a zygote, which develops into one of two types of oocyst; the thin walled, which will excyst and continue the replication cycle within the same host (termed auto-infection), and/or the thick walled, which are shed into the environment (Thompson et al., 2005). Scanning electron microscopy using an *in vitro* cell infection (Human ileocecal adenocarcinoma- HCT8; see below) has given a rough timeline of life cycle progression, with trophozoite development occurring after 24 h of infection, type II merozoites appearing after 48 h of infection and gametes being found after 96 h (Borowski et al., 2010). Several recent studies have suggested that there may be extracellular development (Rosales et al., 1993; Hijjawi et al., 2002, 2004, 2010; Yang et al., 2015; Aldeyarbi and Karanis, 2016a, 2016b, 2016c), and that sexual stages can divide by binary fission (Borowski et al., 2010). Many of these reports show stages bearing little structural similarity however, and have been claimed as being a contamination (Woods and Upton, 2007), while others have seen structural similarities between what groups have termed ‘trophozoites’ in host cell free culture and aged, non-viable sporozoites (Petry et al., 2009). Developing *in vitro* culture methods and higher resolution microscopy techniques, which can support and visualise the parasite for more than two weeks is key to understanding the life cycle and the roles extracellular stages play.

Due to the aforementioned observations, in many pre- and early-millennium publications, *Cryptosporidium* was referred to as an intracellular/extra-cytoplasmic parasite (Lingelbach and Joiner, 1998; Capet et al., 1999; Elliott and Clark, 2000; Arrowood, 2002). However, recent publications have begun using the term ‘epicellular’ to describe *C. parvum* (Thompson et al., 2005; Valigurova et al., 2008; Clode et al., 2015). This is a misleading use of the term, as epicellular pathogens by definition associate with host membrane, but remain outside of the boundaries of the host cell. During infection, the parasite, however, interacts with host cell membrane, causing the cell to attempt to engulf *Cryptosporidium* in membrane material (Elliott and Clark, 2000). As a result, a parasitophorous vacuole forms around the extracellular life cycle stage (either sporozoite or merozoites). The end result is the encapsulation of the parasite in a host-membrane derived vacuole that does not fully enter the host cytoplasm, instead occupying an inter-membranous region of the phospholipid bilayer. The boundaries of a cell can be defined by the outer most aspect of the phospholipid bilayer. Anything within that boundary is intracellular and anything associating with, however closely, but remaining outside is epicellular. *Giardia lamblia* is one such epicellular parasite, utilising a concave surface architecture to ‘suction cup’ onto host cells. By this definition, however, *Cryptosporidium* is now within the borders of the host cell and is thus “intracellular” and not “epicellular”. Most likely this observation is a result of differential resolution in field emission scanning electron microscopy images, and newly advanced microscopy techniques, including atomic force microscopy (AFM), might be able to resolve controversy. Whilst at first glance the images present clearly distinguishable host and parasite, many confocal and electron microscopy images show that this is not the case as such an attachment would not have the effect on host nuclear envelope previously observed (Lingelbach and Joiner, 1998; Miller et al., 2018a).

*Cryptosporidium* parasites have a remarkably streamlined metabolism and reduced biosynthetic pathways (Abrahamsen et al., 2004). *Cryptosporidium* species, specifically *C. parvum and C. hominis*, lack canonical mitochondria, instead having remnant ones, so called mitosomes. These are small (around 100 nm across), circular, double membrane bound structures (Riordan et al., 2003; Keithly et al., 2005), which appear to be functionally reduced mitochondria (Fig. 2b and c) that lack ATP synthesising capability and their own (mitochondrial) genome (Abrahamsen et al., 2004; Hjort et al., 2010; Richardson et al., 2015). However, components of a biochemical pathway (named Iron/Sulpur Cluster assembly; ISC) have been recenty localised in *C. parvum* mitosome, suggesting a functional role of this organelle (Miller et al., 2018b). Other organelles common to apicomplexans, including rhoptries, dense granules and micronemes, are found in the invasive sporozoite and merozoite stages (Petry, 2004). Also visible are the polar rings, which localise to the apical end of the parasite (Petry, 2004). Intracellular stages generally show a loss of the majority of the organelles seen in invasive stages, including the micronemes, rhoptries and dense granules (Beyer et al., 2000). Notable are the presence of dense granules containing amylopectin, ribosomes, a Golgi apparatus, and an endoplasmic reticulum (Keithly et al., 2005).

In light of a collection of morphological and phylogenetic similarities, *Cryptosporidium* has been reclassified from a coccidian to a gregarine (Ryan et al., 2016). Key similarities supporting this new *Cryptosporidium*/gregarine lineage (Liu et al., 2016; Ryan et al., 2016) include completing host-free life cycles, exhibiting large extracellular gamonts, pairing reproductively end to end (syzygy), and changing cell architecture to adapt to diverse environments eg. biofilms, coelom, intestines, soil, and water (Ryan et al., 2016). Other evidence to support this alteration includes the lack of an apicoplast (a remnant plastid present in many other apicomplexans, including *Plasmodium* and *Toxoplasma),* the ability to complete its life cycle in the absence of a host cell *in vitro* (Hijjawi et al., 2004, 2010; Koh et al., 2013, 2014; Aldeyarbi and Karanis, 2016a), and its unique intracellular but extra-cytoplasmic niche (Borowski et al., 2010). It will be necessary for future studies to focus on ultrastructural data in combination with ‘omics (see below) approaches to elucidate further the phylogenetic relationship between *Cryptosporidium* and gregarines, which will allow better understanding of both the nature of the parasites’ oocysts but also their infection strategies.

## 2 *Cryptosporidium* culturing

The lack of a reproducible culture method capable of supporting the parasite life cycle long term (Hijjawi, 2010; Ryan and Hijjawi, 2015; Karanis, 2017) is a major barrier that restricts a greater understanding of this organism. One key process in *C. parvum* culture is the excystation protocol used to prepare oocysts for infection. Numerous laboratories use differing excystation protocols, with the number of excysted sporozoites vs the number of oocysts being used as a measure of viability. It has recently been established that the methods described by Rasmussen et al. provides the highest rate of excystation (Pecková et al., 2016). Another one is the use of primary cell lines and stem cells can provide more accurate *in vitro* models of intestinal infection compared to established epithelial cell lines, allowing higher yields of *C. parvum* extracellular stages. One study has demonstrated that non-carcinoma epithelial cells of the small intestine show similar levels of infection to Madin-Darby Bovine Kidney (MDBK) epithelial cells in terms of percentages of infected cells and an average of just below six infections per cell, nearly double the infections per cell of MDBK cells (Varughese et al., 2014). In another study, *C. parvum* infection was maintained for 120 h using primary human intestinal epithelial cells (Castellanos-Gonzalez et al., 2008), but the short life span and limited availability of primary cells (compared with immortalised cell lines) have prevented wider adoption of this method. Continuous cell lines are limited in their ability to support the parasites life cycle completely and to support continuous development, which tends to plateau after a week in culture (Current and Haynes, 1984; Flanigan et al., 1991; Martinez et al., 1992; Rosales et al., 1993; Upton et al., 1994a; Villacorta et al., 1996). Thus, prolonged development requires host cells that could support the complete life cycle and produce a high yield of both oocyst types to propagate new infections and to continue existing infective cycles.

The first *in vitro* culture system of *C. parvum* utilised endoderm cells of the chorioallantoic membrane of chicken embryos, in which *C. parvum* completed its life cycle between three and eight days (Current and Long, 1983). Substantial developments have been made in cell culture since, utilising several cell types, summarised in Table 1. HCT-8 has been used for most work on *C. parvum* (Hijjawi, 2010; Ryan and Hijjawi, 2015; Karanis, 2017). It has been found that the level of HCT-8 infection can be increased with medium supplementation (Upton et al., 1995). Addition of ascorbic acid, *para*-aminobenzoic acid, folic acid and sodium pantothenate to RPMI-1640 had an enhancing effect on parasite growth (production of high levels of *C. parvum* developmental stages *in vitro*), as did increasing FBS from 5% to 10% v/v in the media. Additional sugar supplements (glucose, maltose, galactose and mannose) and insulin also had a positive effect. The final supplemented medium, consisting of RPMI 1640 with 10% FBS, 15 mM HEPES, 50 mM glucose, and 35 mg of ascorbic acid, 1.0 mg of folic acid, 4.0 mg of 4-aminobenzoic acid, 2.0 mg of calcium pantothenate, 0.1 U of insulin, 100 U of penicillin G, 100 mg of streptomycin, and 0.25 mg of amphotericin B per ml (pH 7.4) supported a ten-fold increase in parasite abundance compared to base RPMI-1640 (Upton et al., 1995). This medium is commonly employed when culturing *C. parvum,* although another medium formulation has been developed which can maintain HCT-8 cells and parasitism for two weeks (Perez Cordon et al., 2007). This formulation consists of RPMI-1640 medium with 10% FBS, pH 7.2 with CaCl_2_ and MgCl_2_ at 1 mM, and also requires that the cells (HCT-8) have no medium renewal for a week prior to infection.

**Table 1:** **Summary of cell lines reported as supporting *C. parvum* replication**. Where multiple cell lines are listed, subsequent data refers to those in bold.

Cell Line/Type	Oocyst Source	Media	Time Maintained	Life Cycle	Reference(s)
Chorioallantoic membrane of chicken embryos (Hubbard broiler and White Leghorn breed)	Naturally infected calf, infected AIDS patient	N/A	8 days	Complete life cycle	Current and Long (1983)
Human foetal lung, Porcine kidney, Primary chicken kidney	AIDS patient	Minimal Essential Media with Earle's Salts, 2% FBS	7 days	Complete life cycle	Current and Haynes (1984)
HT29.74, Clone of colorectal cell line HT29	AIDS patient	RPMI-1640, 10% FBS, 24 mM sodium bicarbonate, 1 mM sodium pyruvate, 20 mM HEPES (for undifferentiated cells), Leibovitz L-15 medium containing 5 mM galactose, 6 mM pyruvate, 1 mM L-glutamine, 20 mM HEPES, antibiotics, 10% dialyzed fetal calf serum (for differentiated cells)	13 days, rapid loss of reinfection after 5 days	Asexual stages only	Flanigan et al. (1991)
Mouse peritoneal macrophages	Naturally infected calves	RPMI-1640, 10% inactivated FBS	3 days	Asexual, few instances of gamonts or oocysts	Martinez et al. (1992)
RL95-2, derived from human endometrial carcinoma	Experimentally infected calves	Dulbecco's modified Eagle's medium and Ham's F12 medium (1:1 ratio), 10% FBS, 10 mM HEPES, 5 μg bovine insulin, 2% NaHCO_3_ (^w^/_v_)	4 days	Complete life cycle	(Rasmussen et al.*,* 1993)
Madin-Darby canine kidney (MDCK)	Infected calves	Minimal Essential Media, 10% FBS	5 days	Complete life cycle	Rosales et al. (1993)
HRT-18 (Human rectal tumour)	Experimentally infected calves	RPMI-1640, 10% Inactivated FBS	6 days	Asexual stages only	(Woodmansee and Joachim, 1983)
Caco-2 (Colorectal adenocarcinoma)	Experimentally infected goats and lambs, AIDS patient with persistent cryptosporidiosis	N/A	5 days	Complete life cycle	(Buraud et al., 1991)
Madin-Darby bovine kidney (MDBK)	Naturally infected calves (Villacorta et al., 1996, Upton et al., 1994a,b)	Minimal Essential Media, 26 mM NaHCO3, 4% FBS (Villacorta), RPMI-1640 with L-glutamine, 10% FBS, 26 mM NaHCO_3_, 15 mM HEPES (Upton)	3 days	Complete life cycle	(Upton et al., 1994a,b; Villacorta et al., 1996)
BALB-3T3, BT-549, Hs700t, HT-1080, RL95-2, HCT-8 (Colorectal adenocarcinoma)	Infected cows	RPMI-1640 with L-glutamine, 10% FBS	3 days	Not described	(Upton et al., 1994a,b)
MRC-5 (lung fibroblast)	Clinical isolate	Medium 199, Earles salts; essential amino acids, L-glutamine, 0.075% w/v sodium bicarbonate, 10% FBS	5 days	Not described	(Dawson et al., 2004)
BS-C-1 (African green monkey kidney cells)	Infected calves	Minimal Essential Media, 2 mM L-glutamine, 10% FBS	3 days	Complete life cycle	(Qi Deng and Cliver, 1998)
COLO-680N	Commercial source (infected cows)	RPMI-1640 with L-glutamine, 10% FBS	8 weeks	Complete life cycle	(Miller et al., 2017)

While long-term culture in a single culture vessel is impossible with the HCT-8 cell line, Hijjawi et al. reported the culture of *C. parvum* in HCT-8 cells for 25 days through sub-culturing (Hijjawi et al., 2001). This was attributed to maintaining the pH of the culture medium between 7.2 and 7.6 by changing the culture medium every two to three days. The success may also be attributed to HCT-8 monolayers up to 67 days old being capable of supporting infection (Sifuentes and Di Giovanni George D., 2007). More recently, *C. parvum* infection was maintained for 120 h using primary human intestinal epithelial cells, which provided a significantly longer infection time compared to 48 h using HCT-8 cells (Castellanos-Gonzalez et al., 2013). Primary cells provide advantages over cancer derived cell lines, such as retaining tissue markers and morphology and are functionally closer to *in vivo* models (Varughese et al., 2014). The disadvantage is their finite lifespan and the resulting need for a continued supply of these cells from animal or human tissues. The morphology of host cells has not been studied in great detail, however some cell lines, which support infection have been reported as having microvillus like extensions (Current and Long, 1983; Flanigan et al., 1991; Upton et al., 1994b). These extensions may play an important role in parasite development, as the observations that microvillus proteins localise to the parasite attachment site (Bonnin et al., 1999). Given the nature of encapsulation by the host cell (Chen et al., 2003; Borowski et al., 2010), host cell morphology may be worth investigating.

Recently, Miller et al. reported the successful propagation of *C. parvum* in the oesophageal squamous cell carcinoma cell line COLO-680N in RPMI-1640 medium supplemented with 10% FBS. COLO-680N cells produced more oocysts and the infection could be maintained for as long as two months without subculturing (Miller et al., 2018a). The presented novel cell culture enables the sustainable, continuous propagation of infectious *C. parvum* oocysts and the systematic investigation of *Cryptosporidium* life cycle. Previously attempts to cultivate *Cryptosporidium* in cell culture were affected by a lack of oocyst production or required sophisticated, expensive equipment and methodologies to support 3D cultures (see below) that are not commonly available to research laboratories (Yin et al., 2010; Karanis and Aldeyarbi, 2011; Striepen, 2013; Muller and Hemphill, 2013; Morada et al., 2016; Heo et al., 2018). The COLO-680N platform does not share these shortcomings. The COLO-680N platform enabled *C. parvum* propagation, the sustainable production of *C. parvum* oocysts, and the investigation of the *C. parvum* biology at a laboratory scale in standard, easy to use 2D tissue cultures with commonly available equipment and particular expertise. Furthermore, their study demonstrated a long-term maintenance of the cell-line and subsequently a prolonged production of oocysts as well, in addition to a first of its kind ability to cryoconserve the parasite for greater long-term storage or transportation, systems both urgently needed by the field (Striepen, 2013; Checkley et al., 2015). The authors also presented for the first time, a collection of additional tools (lipidomics fingerprinting and atomic force microscopy) for investigating the intracellular cell biology and the oocyst morphology and composition of *Cryptosporidium,* which can be incorporated in further studies to provide a better understanding of the infection and life-cycle of the parasite (Miller et al., 2018a).

The need for long term and high yield production of oocysts has led to an interest in 3D or organoid-type culturing systems and organoid models. These systems allow for higher cell densities and subsequent parasite yields, along with allowing long-term parasite propagation. The 3D culture environment may also provide a more accurate model of *in vivo* infection. Early efforts to develop 3D cultures used HCT-8 cells in a low shear microgravity environment, incubated with porcine small intestinal grafts to promote differentiation into cuboidal epithelia and brush border formation (Alcantara Warren et al., 2008). This method allowed the intestinal epithelium to increase parasite numbers for 48 h. However, the infection decreased after this time, accompanied by a reduction in host cell attachment (Alcantara Warren et al., 2008). The method provided a proof of concept, that bioreactor systems can be used to produce *C. parvum* parasites, and with the additional medium supplements, the system may be adapted as a productive culture method. Following this, the culture of *C. parvum* was recently reported using a hollow fibre bioreactor system and maintained for over six months (Morada et al., 2016). The system produced 1 × 10^8^ oocysts per day per mL of culture compared with reported 1 × 10^6^ oocysts produced in traditional 2D culture vessels. Another recent report described the culture of *C. parvum* in a model intestinal system developed using a silk fibre scaffold, which supported infection for two weeks (DeCicco RePass Maria A. et al., 2017). While the method provides a good model of *in vivo* cryptosporidiosis, the use of multiple cell lines including intestinal myofibroblasts and the need for specialist equipment is a large cost barrier for many labs. For long term *Cryptosporidium* culture, the adoption of 3D culture technology by several labs will require a low-cost methodology and simple protocol, which allows routine harvesting of large numbers of oocysts. Even more recently two independent reports have demonstrated propagation of *Cryptosporidium* along with insights on host-parasite interactions in 3D culturing systems. In the first one, *C. parvum* was cultured for up to 27 days using a 3D culture of adult murine colonic explants, while demonstrating new evidence of potential role of the parasite in inducing carcinogenesis in the host (Baydoun et al., 2017). In the second study, the authors demonstrated the *Cryptosporidium* can infect epithelial organoids derived from human small intestine and lung (Heo et al., 2018), while investigating the alterations of the parasite's transcriptome during invasion (see below in ‘omics session). These apparatuses provide a step forward in our understanding of the infection strategies of the parasite and production of oocysts in animal-free models, but they have their limitations in both investigating the parasite's invasion in real time, but also in utilising these in large-scale drug screening investigations.

Despite these advances, there has also been considerable interest in propagating the parasite in the absence of host cells. This method would offer considerable advantages compared to host cell culture removing the need to purify the parasite from host cell material. The first report of *in vitro* culture was by Hijjawi in 2004, who described the entire life cycle of *C. parvum* in host cell free culture (Hijjawi et al., 2004). Utilising a modified version of RPMI 1640 with supplements and coagulated calf serum, the authors observed stages from inoculated sporozoites through to sporulated oocysts, along with additional extracellular stages. Previously, novel extracellular stages had been purified from the guts of infected mice (Hijjawi et al., 2002). Unfortunately, the results observed were not readily reproducible by other groups (Girouard et al., 2006; Woods and Upton, 2007). Woods and Upton observed that the figures reported as being ‘extracellular gamont like stages’ were more likely to be fungal contaminants (Woods and Upton, 2007) given the similar morphology and the lack of antifungals in the culture medium (Hijjawi et al., 2004). Previous reports (Karanis et al., 2008) have demonstrated the capability of maintaining the merozoite stages of *Cryptosporidium*, but the authors were not able to further culture the parasite. As discussed earlier, two groups have determined that alleged ‘merozoites’ were in fact aged sporozoites (Petry et al., 2009; Matsubayashi et al., 2010). A recent study using the described maintenance medium (Hijjawi et al., 2004) to culture *C. parvum* showed stages using scanning electron microscopy (Yang et al., 2015). Only stages from inoculated oocysts to type-I merozoites were described, and there were no cases of either sexual stages or the previously described ‘extracellular gamont like stages (Hijjawi et al., 2004) in culture. Lately, Aldeyarbi and Karanis described the complete life cycle of *C. parvum* in the absence of host cells using cell free maintenance medium Express Five™ serum free medium (Aldeyarbi and Karanis, 2016a, 2016b, 2016c).

In summary, cell-free culture has its appeals and needs to be investigated further as an alternative to current commercial animal sources, but what is urgently needed is an accurate *in vitro* model of infection. Currently, the COLO-680N platform can provide a robust 2D culturing system, which allows a more in-depth examination of the host-parasite relationship as well as a life-cycle wide examination of parasite metabolic processes (Miller et al., 2018a). This culturing platform will enable the investigation into the genetic, cellular, biochemical, immunological and metabolomic alterations of *Cryptosporidium* and its host and to develop new drug screening platforms based on this cell line. Using similar tactics (Miller et al., 2018a), future research should also focus on exploring other potential cell systems that could be used in addition to animal models or organoid-type culturing to spearhead our understanding of the disease and the life cycle of *Cryptosporidium*.

## 3 *Cryptosporidium* ‘omics and *in vitro* research

To understand the biology of the parasite, we survey the current knowledge of *in vitro Cryptosporidium* ‘omics, focusing on gene expression profile upon excystation and host infection and to which degree these changes may influence the host and/or govern infectivity. It is understood that upon infection, both host cells and parasites undergo major remodelling in gene regulation such as the induction of factors involved in the host immune response or the parasite invasion process, respectively. However, it is clear that the dynamics of gene expression extend beyond these pathways and this is why a great fraction of recent studies have focused on identifying RNAs or proteins with altered expression during the infection process, by examining the *status quo* of *Cryptosporidium* undergoing the excystation process and/or while interacting with its host. For *C. parvum,* the implementation of transcriptomics and proteomics studies has proven very challenging, due to the difficulty of isolating intracellular stages of infection and the lack of a gene engineering platform. However, recent advances, such as the development of *in vitro* models (see section above) as well as the development of novel gene-editing tools (Vinayak et al., 2015; Pawlowic et al., 2017), hold great promises for this research field.

Genomic sequence analysis yields much biological information and allows to compare genome features of different parasites. The first complete genome sequence came from a *C. parvum* isolate subtype IIa, using automated capillary sequencing (Abrahamsen et al., 2004). The sequence was later improved by next generation sequencing and the revised annotation identified 3805 protein-coding genes (Isaza et al., 2015). Further sequence comparison between the type II a isolate (IOWA) and another subtype IId isolate from El Beheira Province, Egypt, showed that significant genomic differences could exist between genomes from the same species and that biological differences between isolates could be attributed to sequence polymorphism (Feng et al., 2017). A collection of genomic and transciptomic data are currently available in *Cryptosporidium*-dedicated database at the CryptoDB (http://cryptodb.org/cryptodb/) (Heiges et al., 2006). These analyses established that the *C. parvum* genome lacks apicoplast and mitochondrial genes and has fewer genes encoding metabolic function and variant surface proteins compared to other apicomplexan parasites (Abrahamsen et al., 2004). Notably, the *C. parvum* genome holds very few introns (only 5% of genes have introns), and the parasite encodes therefore few protein isoforms. Since then, additional *Cryptosporidium* genomes (*C. muris, C. andersonii* and *C. ubuiquitum*) and strains have been sequenced, showing genetic variation and composition in the various species, and in extreme cases, further genetic streamline (Liu et al., 2016).

To further understand the biology of the parasite, the use of proteomics techniques is also important. Siddiki et al., for example, used mass spectrometry based BLAST (MS BLAST) to identify *C. parvum* proteins from frozen sporozoites pellets isolated from lamb faecal samples. They separated the total proteins by 1D-SDS-PAGE and analysed 20 slices by 2D-n-LC MS/MS (Siddiki and Wastling, 2009). Applying this approach, the authors identified 84 *Cryptosporidium*-specific proteins; a third being previously hypothetical and another third being involved in protein biosynthesis. In another study, Snelling et al. also used a mass spectrometry approach, but rather than solely analysing the proteome of *Cryptosporidium*, they also tried to detect proteins which exhibited differential expression in excysted sporozoites compared to non-excysted sporozoites (Snelling et al., 2006). Their proteomics’ analyses revealed the expression of 26 proteins, which were significantly induced following excystation, including ribosomal proteins, metabolic enzymes and heat shock proteins. Interestingly, three of the identified proteins were apicomplexan-specific and five were *Cryptosporidium*-specific. As suggested by the authors, all identified proteins could be involved in vital step of the pathogenesis. However, it remains to determine whether the same response is elicited when *Cryptosporidium* is actually in contact with the host or internalised.

Ultimately, it is the flux of metabolites and the dynamics between proteins from parasite and host, which govern their biological interaction, leading to the susceptibility or not of the host cell or organism. The regulation of gene expression in *C. parvum* in relation with its host, rather than in the process of excystation, has been the focus of many studies. Some of these studies concentrated on the alterations of the parasite as it follows its life cycle within the host, while others tried to understand the interactions between the two organisms. Mauzy et al., carried out a large-scale expression analysis *in vitro* on 3281 *Cryptosporidium* genes using RT-PCR, looking at seven post-infection time points from 2 to 72 h in HCT-8 cells (Mauzy et al., 2012). Their work revealed numerous *Cryptosporidium* genes that share the same pattern of expression. However, genes within a cluster did not map to the same chromosomal location. Overall, they identified nine clusters, which were associated with 18 categories of cellular processes, including transcription, translation, and metabolism. This work suggests that the bulk of translation and turnover does not occur immediately after anchorage of the parasite to the host cell, but in a later stage, when the sporozoite has developed to trophozoites. Interestingly, genes present in cluster six are mostly involved in transcription and RNA-associated event (approximately 17%) as well as translation and protease related processes (approximately 13%). This cluster exhibits a peak of expression at 2 h’ post infection. Transcripts in cluster nine are involved in translation (24%) and protease related processes (5%), but less so in transcription and RNA-associated events (13%), and show a peak of transcription at 6 h post infection. Oberstaller et al. took a different outlook on the Mauzy et al. study. They grouped the same genes according to their pattern of expression, but used different clustering criteria to generate 200 clusters (Oberstaller et al., 2013). They then searched for conserved and over-represented DNA motifs in the upstream promoter region of these genes and identified 25 DNA motifs, which were further classified into 11 families. While some of these DNA motifs are similar to the palindromic apicomplexan AP2 (ApiP2) binding site, known as a transcriptional regulator (Mullapudi et al., 2007; Yuda et al., 2010), many of the motifs are uncharacterised. Though some are likely to modulate the outcome of infection by controlling gene expression, it is hard to draw any conclusion at this stage. To our knowledge there is no further study, which validates transcription factors or proteins binding to these putative DNA motifs.

In another study by Wang et al., rather than measuring changes of the full transcriptome, the authors examined the transcripts with very low protein coding potential in infected epithelial cells. They showed that this particular class of transcripts exhibited distinct gene expression patterns, at different parasitic development stages, with some of which are found at higher level in the sporozoites (Wang et al., 2017). Many of the latter were discovered in the nuclear extract of infected cells, possibly to modulate host cell gene expression. The implication of these observations remains speculative, but shows that *Cryptosporidium* has evolved a complex and diverse strategy to invade and control its host.

While it is essential to identify changes in expression, knowing if some of the co-regulated products interact or form complexes, give the true picture of biological interaction (Reid and Berriman, 2013). Currently, we know next to nothing about which proteins interact between parasites/host, since information retrieved from host-parasite protein-protein interactions in *Cryptosporidium*-infected hosts is very limited. It currently relies on specific sequence/protein conservations and there is a need of a large-scale analysis looking not only at co-regulation between parasite and host, but also exploring the shaped microbiome and metabolome. As thus, a combination of experimental infections in animals and or organoids (see above) followed-up by a combination metagenomics, transcriptomics (Heo et al., 2018) and metabolomics (using mass spectrometry and/or ^1^H Nuclear Magnetic Resonance-based) would allow a better understanding on the parasites’ interactions with their hosts and determination of the factors that underlie the variable clinical consequences of *Cryptosporidium* infection.

## 4 *Cryptosporidium* drug screening and potential treatments

While *Cryptosporidium* species are globally observed, they have the most devastating effect in less developed countries, and thus the current strategy has been focused on drug repurposing (use of existing medications) rather than the development of rational drug design-a costly tactic. To date, nitazoxanide is the only FDA approved treatment against infection by *Cryptosporidium.* However, as highlighted previously, it remains inefficient especially on immunocompromised patients such as HIV-positive patients. Therefore, the search for a drug more appropriate for a large spectrum of patients goes on.

Compounds used versus many apicomplexan parasites are unsuitable against *Cryptosporidium* infection, since the parasite lacks the apicoplast organelle, the citrate cycle and the cytochrome-based respiratory chain. Nonetheless, other metabolic pathways are still present and could be used as potential drug targets. With this approach in mind, Besoff and co-authors screened the Medecines for Malaria Venture (MMV) Open Access Malaria box, a collection of 400 compounds, by measuring growth inhibition of *Cryptosporidium* within HCT-8 infected cells (Bessoff et al., 2014). The allopurinol scaffold (0.12 μM) and the quinolinol scaffold (0.054 μM) showed the best activity against *C. parvum* infection. Other studies have been restricted to specific groups of compounds. For instance, studies have used the benzimidazole nucleus, as it forms the core of many antiparasitic, antihelminthic, antifungal and anti-inflammatory drugs. This observation prompted Graczyk's group to examine the effect of 11 benzimidazole derivatives on *C. parvum* -infected HCT-8 cell using an immunoassay approach with rat anti-*Cryptosporidium* polyclonal sera (Graczyk et al., 2011). Nine out of 11 benzimidazole compounds tested showed reduced volume of developmental stages of *C. parvum in vitro*. Nonetheless, the mechanisms of inhibition were not clear and potential side-effects to the host's liver have been hinted at.

To step up the search for suitable treatments that benefit all sufferers, a more systematic approach is necessary. Thus, the field has seen the emergence of cell-based high-throughput assays designed to screen large panel of existing drugs. For example, Bessoff et al. performed a high throughput screening, on *C. parvum*-infected HCT-8 cells, testing the National institute of Health library of compounds, a collection of 723 FDA-approved drugs (Bessoff et al., 2013). They identified pyrimidine analogues and statin drugs as promising anti-*Cryptosporidium* compounds-showing over 80% inhibition of *Cryptosporidium* at below 10 μM concentration. Refining their analysis using structure activity relationship (SAR), they isolated itavastatin, an inhibitor of human 3-hydroxy-3 methyl-glutaryl-coenzyne A reductase. In a parallel study, Love et al. used a methodology modified from Bessof et al. (Bessoff et al., 2013) and screened 78,942 compounds to identify potential anti-cryptosporidial drugs (Love et al., 2017). Their bioactive hits for *C. parvum* were also counter-screened against *C. hominis*. While a few potent compounds against *Cryptosporidium* were identified, this type of phenotypic screen has limitations. It is difficult to identify whether observed effects are due to inhibition of the host or inhibition of *Cryptosporidium* activity. As a result, most of the drugs initially retained after the screen, exhibited a limited therapeutic index. Clofazimine, on the other hand, was taken forward and was further tested in a killing activity assay (treatment of *in vitro* cell cultures, followed by compound washout), which showed that the parasite growth was greatly reduced (>70–75%) at EC_50_ of 15 nM. More strikingly, single-dose treatment on mice also effectively reduced oocyst shedding. This study therefore established Clofazimine as a potential repurposing candidate for the treatment of cryptosporidiosis. Lastly, Manjunatha et al. screened 6220 compounds against *C. parvum* in an infection assay in HCT-8 and found that imidazopyrzynes and pyrazolopyridines exhibited sub-micromolar cellular activity against *Cryptosporidium* (Manjunatha et al., 2017). The pyrazolopyridine KDU731 came up as a potential anti-cryptosporidial drug candidate that is active against both *C. parvum* and *C. hominis*. Additionally, unlike nitazoxanide, KDU731 demonstrated *in vivo* efficacy in immunocompromised mice.

Beside these large-scale studies, which are expensive and often require several replicates of screening, *in silico* approaches offer the advantage that they predict structure and biological characteristics of proteins. Shrivastava et al. for example, used CryptoDB database (Heiges et al., 2006) to find novel drug targets using the parasites predicted proteome (Shrivastava et al., 2017). Roughly, 50% of proteins in both *C. hominis* TU502 and *C. parvum* IOWA in the CryptoDB database are hypothetical or unnamed. From these, 105 proteins that were present in both species, were searched for their uniqueness via BLAST analysis, with the reasoning these might be potential drug target. Such approach revealed a unique hypothetical protein in *C. hominis* genome TU502HP. The authors, however, did not describe the nature of this uniqueness and simply states that it bears 91% sequence identity with *C. parvum* (CryptoDB: cgd2_2550) with functional similarity with human transporting 3. A docking study reported *N*-(3-chlorobenzyl)ethane-1,2-diamine as the best inhibitor in terms of docking score and binding energy. In this instance, the approach, however, is restricted to one *Cryptosporidium* species, namely *C. hominis*, as it purposely selects for a unique trait/gene target. However, while this *in silico* is innovative, the inhibitor/target interaction described in this study remains to be validated experimentally. Because *Cryptosporidium spp* interaction with its host(s) is complex, drug target identification requires *in vitro* and *in vivo* validation in order to identify compounds suitable for follow-up testing. *In vitro* (cell lines) models have to be considered in the context of drug absorption and metabolism, in animals and humans and it therefore necessary to take a multi-validation approach as *in vitro* and *in vivo* models are likely to show only partial functional overlap. For example, earlier work by Culshaw et al., have suggested that IFN-gamma protects against many protozoan parasites including *Cryptosporidium* species (Culshaw et al., 1997). Transgenic mice models (e.g. IFN-gamma R-KO) were shown to survive gavage by large number of oocysts (2000 oocysts per animal) and achieved high rate of amplification (von Oettingen et al., 2008). *In vitro* studies in cell cultures have also shown a direct inhibitory action of IFN-gamma on *C. parvum* infected human enterocytes, but this was dependent on the cell type (Caco-2 and HT-29, but not HT-4) and the amount of time elapsed between infection and treatment with INF-gamma. While *in vivo* studies highlight the role played by IFN-gamma in *C. parvum* infection in mice (Lykens et al., 2010), the outcome is less clear in human infections (Janssen et al., 2002). Taken together, this shows that additional studies are essential to further define the role of cytokines in the immune response to *Cryptosporidium* infection. For example, the transgenic INF-gamma mouse model has been used in combination with genetic manipulation of *C. parvum* (UGA1 strain) (Vinayak et al., 2015; Pawlowic et al., 2017)to rank the safety and efficacy of Bumper Kinase Inhibitors (BKIs) against *C. parvum* Calcium-Dependent Protein Kinase (CDPK1), showing that BKI 1369 offers positive prospects of future therapy for infected patients.

In summary, a lot of effort has been invested in the search for new treatment against cryptosporidiosis, which may see the emergence of new drugs on the market. Currently, clofazimine and TU502HP inhibitors are both potential candidates, while clofazimine, as a repurposed-drug, may not need approval for new handlings. In parallel, there is an urgent need for the development of high-throughput drug screening platform for both *C. parvum* and *C.hominis* that will enable both the identification of anti-*Cryptosporidium* compounds but also chemical biology approaches to elucidate *Cryptosporidium* biology. The long-term cultivation culturing system (Miller et al., 2018a) along with transfection (e.g. using the CRISPR/Cas9 system) protocols (Vinayak et al., 2015) that will allow the visualisation of fluorescent parasites will enable monitoring *Cryptosporidium* replication and, hence identification of additional anti-parasitic drug candidates.

## 5 Future work

While substantial improvements have been made in understanding *Cryptosporidium* parasites and their biology, much progress remains to be made. Recent successes in the fields of long term culturing (Heo et al., 2018; Miller et al., 2018a) along with the establishment of the CRISPR/Cas9 technology (Vinayak et al., 2015) may facilitate a number of these required developments that are urgently needed, especially in the areas of drug treatment and vaccine development. The cell biology of *Cryptosporidium* infection has been widely documented using various microscopy techniques, however live cell imaging allowing progress to be tracked in real time is still lacking. In addition, the molecular basis of the life cycle changes has yet to be discerned. In the future, it may be possible to routinely transfect *Cryptosporidium* and maintain it in a long-term culturing platform (Miller et al., 2018a) to identify crucial functional genes, their roles and possible drug and vaccine targets. In addition, while a great number of *Cryptosporidium* predicted proteins are currently of unknown function, knockout studies and monitor *Cryptosporidium* physiology/behaviour during infection, may also provide some insight into their function. Being able to maintain stable subtypes of the parasite, consider interactions of the parasite and potential drugs and to assess the relationships between genotype and pathogenicity, it would be possible to develop effective treatments and vaccines against cryptosporidiosis.

## Appendix A Supplementary data

Data Profile Data Profile
